# Latent profile analysis of basic psychological need satisfaction and frustration in community-dwelling older adults and its associated factors

**DOI:** 10.3389/fpsyg.2026.1832763

**Published:** 2026-06-03

**Authors:** Chen Yuhang, Zeng Rong, Gan Xueqing, Feng Jing, Zhou Li, Deng Zhaoxia, Tan Yuanyuan, Deng Li, Yang Qian

**Affiliations:** 1 Chengdu Medical College School of Nursing, Chengdu, Sichuan, China; 2 The First Affiliated Hospital of Chengdu Medical College‌, Chengdu, Sichuan, China; 3 Bailianchi Community Health Service Center‌, Chengdu, Sichuan, China; 4 Xihanggang Community Health Service Center, Chengdu, Sichuan, China; 5 Shaheyuan Community Health Service Center, Chengdu, Sichuan, China; 6 Wannian Community Health Service Center, Chengdu, Sichuan, China

**Keywords:** basic psychological need frustration, basic psychological need satisfaction, community, health, older adults

## Abstract

**Objective:**

To explore the latent profile types of basic psychological need satisfaction and frustration in community-dwelling older adults and to identify their associated factors, thereby providing evidence for the development of targeted mental health interventions.

**Methods:**

A convenience sampling method was used to recruit 430 older adults living in two communities in Chengdu from October to December 2025. Data were collected using a general information questionnaire, the Chinese version of the Basic Psychological Need Satisfaction and Frustration Scale, the Satisfaction with Life Scale (SWLS), the Short Form of the Depression Anxiety Stress Scales (DASS-21), and the Perceived Social Support Scale (PSSS). Latent profile analysis was conducted to identify subgroups of basic psychological need satisfaction and frustration. Logistic regression analysis was performed to examine the associated factors of different latent profiles.

**Results:**

The latent profile analysis identified four distinct profiles: the Basic Psychological Need Deficiency Type (8.1%), the Basic Psychological Need Satisfaction Type (46.3%), the Basic Psychological Need Balance Type (33.3%), and the Basic Psychological Need Contradiction Type (12.3%). Logistic regression analysis showed that monthly income, living situation, health status, satisfaction with life, depression–anxiety–stress levels, and perceived social support were significant predictors of profile membership.

**Conclusion:**

There is significant heterogeneity in basic psychological need satisfaction and frustration in community-dwelling older adults. Tailored and stratified mental health interventions should be implemented based on the characteristics of different profiles to enhance basic psychological need satisfaction and promote active aging.

## 1 Introduction

As the aging process of China’s population accelerates and the size of the older adult population continues to expand, community-dwelling older adults have become the majority of the elderly population. According to data from the seventh national census, China’s population aged 60 and over has exceeded 260 million, with the degree of aging deepening (National Health Commission of the People’s Republic of China, Department of Elderly Health, 2021). The “14th Five-Year Plan” for the Development of National Undertakings for the Aged and the Construction of the Elderly Care Service System explicitly proposes to strengthen mental health services for the elderly, promote active aging, and improve the quality of life of older adults in the community (State Council of the People’s Republic of China, 2021). Old age is not only a crucial stage characterized by the gradual decline of physiological functions but also a period highly concentrated with psychological adaptation and social role transitions (Carstensen, 2021). How to promote the mental health of community-dwelling older adults, enhance their quality of life, and contribute to the realization of the active aging strategy has become an urgent and important issue in the fields of public health and geropsychology.

Basic Psychological Needs Theory, a sub-theory of Self-Determination Theory, is widely used in psychological research. Basic Psychological Needs Theory posits that humans have three basic psychological needs: autonomy, relatedness, and competence. Correspondingly, there are three instances of basic psychological need satisfaction and three instances of basic psychological need frustration. If these needs are consistently met, it helps individuals maintain mental health and enhance well-being; if basic psychological needs are continually thwarted, it can easily induce psychological disorders and inner distress (Vansteenkiste et al., 2020; Ryan and Deci, 2017). In old age, the developmental context for community-dwelling older adults undergoes significant changes. Retirement may diminish their experience of autonomy in social decision-making and self-direction. Declining physical function and increased chronic diseases can reduce their sense of competence, making them feel limited in their ability to engage in daily activities and social participation. The shrinking of social networks and the loss of relatives and friends may weaken relational support, affecting their sense of belonging and emotional connection (Hajek and König, 2020; Kriebs, 2024; Andersson Hammar et al., 2024). These factors can lead to varying degrees of fluctuation in community-dwelling older adults’ autonomy, competence, and relatedness, causing the basic psychological need satisfaction and frustration (BPNSF) to exhibit more complex characteristics.

Current research has primarily adopted variable-centered approaches, focusing on the single variable of either basic psychological need satisfaction or frustration, and lacking studies that simultaneously examine both dimensions. This may overlook the potential co-existence and interaction between basic psychological need satisfaction and frustration (Li, 2023; Liu et al., 2025a,b). Recent evidence suggests that basic psychological need satisfaction and frustration are not opposite poles of a single continuum, but rather relatively independent dimensions that may co-occur within individuals and form complex psychological patterns (Vansteenkiste et al., 2020). Despite these theoretical advances, empirical studies simultaneously examining both dimensions remain limited, particularly among older adult populations. Moreover, variable-centered approaches assume population homogeneity, which may obscure meaningful subgroup differences. Latent profile analysis (LPA), as a person-centered approach, allows for the identification of distinct subgroups based on individuals’ response patterns, thereby capturing heterogeneity that cannot be detected through traditional methods (Yi et al., 2020). By simultaneously considering both basic psychological need satisfaction and frustration, LPA may provide a more nuanced understanding of psychological functioning in community-dwelling older adults.

To address these gaps, this study aims to employ latent profile analysis to identify distinct subgroups of community-dwelling older adults based on their patterns of basic psychological need satisfaction and frustration, and to explore the sociodemographic and psychosocial factors associated with each profile. Unlike traditional variable-centered research, this study simultaneously considers both dimensions to capture the complex co-occurrence patterns that may exist within individuals. It highlights the unique research value of simultaneously examining need satisfaction and frustration from a person-centered perspective if only satisfaction or frustration were considered in isolation, a subgroup characterized by the co-occurrence of high levels on both dimensions would be overlooked. By revealing the heterogeneity in basic psychological need experiences among community-dwelling older adults, the findings are expected to provide empirical evidence for developing stratified and targeted mental health interventions, ultimately contributing to the promotion of active aging.

## 2 Methods

### 2.1 Study participants

Using a convenience sampling method, community-dwelling older adults from two communities in Chengdu were selected as study participants from October 2025 to December 2025. Inclusion criteria were: (1) aged 60 years or older; (2) permanent residents who had lived in the project area for more than 6 months in the 12 months prior to the survey; (3) provided signed informed consent. Exclusion criteria were: (1) individuals with severe organic diseases or mental disorders who were unable to cooperate with the survey; (2) individuals with severe physical disabilities who were unable to complete the questionnaire independently. In this study, there were 8 items in the general information questionnaire, 6 dimensions in the Basic Psychological Need Satisfaction and Frustration Scale, 1 dimension in the Satisfaction with Life Scale, 3 dimensions in the Depression Anxiety Stress Scales, and 4 dimensions in the Perceived Social Support Scale. Referring to the sample size estimation method for multivariate analysis (Ni et al., 2010) and considering a 5%–10% dropout rate, the final sample size was determined to be 430 cases. This study was approved by the Ethics Committee of Chengdu Medical College (Ethics Approval No: 2024NO.107), adhered to the Declaration of Helsinki. All data were used solely for this research, and all participants had the right to withdraw from the study at any time. All participants were informed about the study and participated voluntarily.

The final valid sample consisted of 430 community-dwelling older adults, with a mean age of 67.07 ± 10.97 years. In terms of sex, there were 175 males (40.7%) and 255 females (59.3%). Regarding education level, 190 participants (44.2%) had primary school education or below, 110 (25.6%) had junior high school education, 69 (16.0%) had senior high school or technical secondary school education, and 61 (14.2%) had college education or above. For monthly income, 197 participants (45.8%) earned less than 3,000 RMB, 103 (24.0%) earned 3,000–5,000 RMB, and 130 (30.2%) earned more than 5,000 RMB.

### 2.2 Survey instruments

This study covariate selection was strictly guided by Basic Psychological Needs Theory and robust geropsychological evidence, including demographic factors as determinants of access to need-satisfying resources (Tang et al., 2020), health-related factors as prerequisites for autonomy and competence (Hansson et al., 2019), and psychosocial factors as direct correlates of need satisfaction and frustration (Obioha et al., 2025).

#### 2.2.1 General information questionnaire

This questionnaire was designed by the researchers and included 15 items: age, sex, education level, monthly income, marital status, living situation, number of chronic diseases, and health status.

#### 2.2.2 Chinese version of the basic psychological need satisfaction and frustration scale

This scale was adapted into Chinese by Liu et al. (2025a,b). The questionnaire comprises two subscales: Basic Psychological Need Satisfaction and Basic Psychological Need Frustration, encompassing six dimensions with a total of 24 items. The total score ranges from 24 to 120. Higher scores indicate greater satisfaction of psychological needs and higher levels of psychological need frustration, respectively. This scale has been validated and used among Chinese older adults. In this study, the Cronbach’s *α* coefficient for this scale ranged from 0.921 to 0.954.

#### 2.2.3 Satisfaction with life scale (SWLS)

Adapted into Chinese by Wang et al. (2008), this scale assesses an individual’s quality of life. This scale has been validated and used among Chinese older adults. It consists of one dimension with 5 items. Higher scores indicate greater life satisfaction. In this study, the Cronbach’s α coefficient for this scale was 0.948.

#### 2.2.4 Short form of the depression anxiety stress scales (DASS-21)

This scale was translated into Chinese by Gong et al. (2010). It consists of three subscales (depression, anxiety, and stress), with a total of 21 items. Each item is rated on a 4-point Likert scale ranging from 0 (does not apply) to 3 (always applies). The score for each subscale is multiplied by 2 to obtain the final scale score. Higher scores indicate more severe levels of depression, anxiety, and stress. This scale has been validated and used among Chinese older adults. In this study, the Cronbach’s *α* coefficient for this scale was 0.94.

#### 2.2.5 Perceived social support scale (PSSS)

Adapted into Chinese by Jiang (2001), this scale includes three dimensions: family support, friend support, and support from other significant others, with a total of 12 items. The total score ranges from 12 to 84. Higher scores indicate greater perceived social support among participants. This scale has been validated and used among Chinese older adults. In this study, the Cronbach’s α coefficient for this scale was 0.931.

### 2.3 Procedure

This study used Questionnaire Star, an online survey platform, to distribute electronic questionnaires and collect data. First, the researchers explained the study purpose, methods, confidentiality measures, voluntary participation principle, and other ethical considerations in detail to community administrators. After obtaining approval from the university’s ethics committee and informed consent from the administrators, electronic questionnaire links and electronic informed consent forms were distributed to eligible community-dwelling older adults. All questionnaires were administered in a fixed order to all participants. The order was as follows: (1) the general information questionnaire, (2) the Chinese version of the Basic Psychological Need Satisfaction and Frustration Scale, (3) the Satisfaction with Life Scale, (4) the Short Form of the Depression Anxiety Stress Scales, and (5) the Perceived Social Support Scale. All questions were set as mandatory to prevent missing responses, and IP address restrictions were applied to ensure that each device could submit the questionnaire only once, thereby guaranteeing data uniqueness. After data collection, a data validation team consisting of two uniformly trained researchers screened valid responses based on predefined criteria: questionnaires with abnormal completion time (<3 min or >20 min), patterned responses (e.g., consecutive identical choices), or logical contradictions were excluded to ensure data authenticity and validity. There is no systematic selection bias in this study. A total of 435 questionnaires were distributed in this survey. After rigorous quality control and screening, 430 valid questionnaires were finally collected, with five invalid questionnaires excluded, yielding a valid response rate of 98.8%.

### 2.4 Statistical analysis

Mplus 8.3 software was used to conduct latent profile analysis. Models with one to five profiles were estimated sequentially. The following three types of indices were used to determine the optimal model fit: (1) Information criteria: Akaike Information Criterion (AIC), Bayesian Information Criterion (BIC), and sample-size adjusted BIC (aBIC). Smaller values indicate better model fit. (2) Classification accuracy: Entropy was used to evaluate the precision of classification. Entropy values closer to 1 indicate more accurate classification; an Entropy value of 0.8 suggests that classification accuracy exceeds 90%. (3) Likelihood ratio tests: The Lo–Mendell–Rubin adjusted likelihood ratio test (LMRT) and the Bootstrap likelihood ratio test (BLRT) were used to compare the fit differences between models with k-1 classes and k classes. A *p*-value < 0.05 indicates that the model with k classes fits significantly better than the model with k-1 classes.

Data analysis was performed using SPSS 27.0 software. Normally distributed continuous variables were expressed as mean ± standard deviation, and comparisons between groups were performed using one-way analysis of variance. Non-normally distributed continuous variables were expressed as median and interquartile range, and comparisons between groups were performed using the Kruskal–Wallis H test. Categorical or ordinal data were summarized as frequencies and percentages, and group differences were evaluated using the chi-square test or the Kruskal–Wallis H test, as appropriate. Multivariate analysis was conducted using logistic regression models. No data standardization or transformation was applied; all variables were retained in their original metrics for latent profile analysis and multinomial logistic regression.

First, LPA was performed to identify latent profiles based on the six dimensions of the BPNSF, with each dimension score treated as a continuous indicator variable. LPA relies on the assumption of conditional normality and local independence: observed indicator variables are assumed to follow a normal distribution within each latent profile (rather than across the whole sample), and indicators are locally independent within each profile. The optimal four-profile solution was determined based on fit indices, entropy value and high average posterior classification probabilities, indicating satisfactory classification quality and adequate model fit. Subsequently, with latent profile membership as the dependent variable, univariate analysis and multinomial logistic regression were conducted to explore the associations of demographic, health, and psychosocial factors with latent profile membership. A *p*-value < 0.05 was considered statistically significant.

## 3 Results

### 3.1 Common method Bias

Harman’s single-factor test was conducted, revealing eight factors with eigenvalues greater than 1. The first factor accounted for 38.982% of the total variance, indicating that no significant common method bias was present in this study.

### 3.2 Latent profile analysis of the basic psychological need satisfaction and frustration (BPNSF) in community-dwelling older adults

Latent profile analysis was performed on the scores of the six dimensions of basic psychological need satisfaction and frustration among 430 community-dwelling older adults. Models with one to five profiles were estimated sequentially. The fit indices for the latent profile models are presented in Table 1. When the model had one class, it assumed that the study population was homogeneous and had no latent subgroups, and therefore could not explain individual differences among the observed variables, resulting in the poorest model fit. The two-profile and three-profile models had lower Entropy values and higher AIC, BIC, and aBIC values compared to the four-profile model. Although the 5-class model showed slightly lower AIC, BIC, and aBIC values, the improvement compared with the 4-class model was marginal. In addition, the entropy of the 4-class model was the highest (0.964), indicating better classification accuracy. Furthermore, the additional class in the 5-class solution did not provide a clearly distinct or theoretically meaningful profile, but rather represented a further subdivision of existing groups. Considering classification accuracy, interpretability, and the principle of parsimony, the 4-class model was selected as the optimal solution. To further verify the reliability of the latent profile results, the average posterior probabilities for the four profiles were calculated, yielding values of 97.9%, 99.4%, 96.0%, and 99.7%, respectively, all exceeding 85% (Nylund-Gibson and Choi, 2018). This confirms the high reliability of the four-profile model (Table 2).

**Table 1:** Model fit indices for the latent profile analysis of BPNSF in community-dwelling older adults.

Profiles	AIC	BIC	aBIC	LMRT	BLRT	Entropy	Proportions
1	7380.008	7428.773	7390.692	—	—	—	1
2	6332.144	6409.356	6349.061	0.000	<0.001	0.952	0.714/0.286
3	5556.416	5662.074	5579.565	0.017	<0.001	0.948	0.219/0.514/0.267
4	**5077.896**	**5212.001**	**5107.278**	**0.021**	**<0.001**	**0.964**	**0.081/0.463/0.333/0.123**
5	4693.599	4856.150	4729.214	0.013	<0.001	0.950	0.081/0.144/0.285/0.366/0.124

AIC, Akaike Information Criterion; BIC, Bayesian Information Criterion; aBIC, sample-size adjusted BIC; LMRT, Lo–Mendell–Rubin adjusted likelihood ratio test; BLRT, Bootstrap likelihood ratio test. Entropy measures classification accuracy (values closer to 1 indicate better accuracy). Proportions are class sizes relative to total sample. The 4-class model was selected as optimal based on a combination of information criteria, entropy, and theoretical interpretability. Bold values indicate fit indices for the best-fitting model.

**Table 2:** Average posterior probabilities for the four latent profiles.

Type	Class 1	Class 2	Class 3	Class 4
Class 1	**0.979**	0.000	0.021	0.000
Class 2	0.000	**0.994**	0.006	0.000
Class 3	0.007	0.027	**0.960**	0.006
Class 4	0.000	0.000	0.003	**0.997**

Values on the diagonal represent the average posterior probability of being assigned to the correct latent profile. Off-diagonal values represent the average probability of being assigned to other profiles. Values in bold represent the average posterior probability of correct classification for each latent profile in this study. All diagonal values exceeded 0.96, indicating excellent classification accuracy.

**Figure 1:**
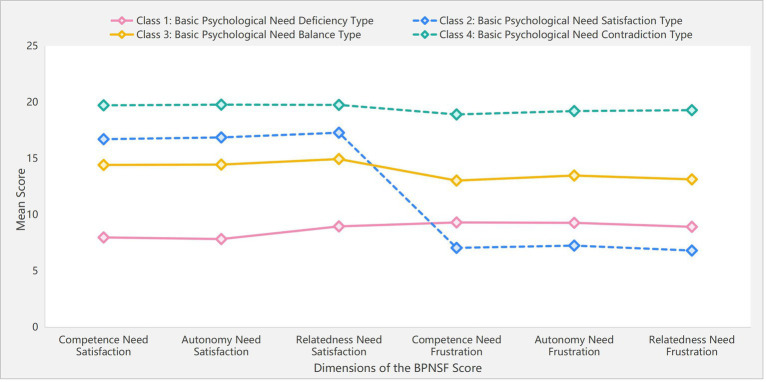
Latent profile of BPNSF in community-dwelling older adults. Values are mean scores of each latent profile on the six dimension. Basic psychological need deficiency type (Class 1): uniformly low satisfaction and moderate frustration; basic psychological need satisfaction type (Class 2): high satisfaction and low frustration; basic psychological need balance type (Class 3): moderate levels of both satisfaction and frustration; basic psychological need contradiction type (Class 4): high levels of both satisfaction and frustration.

Based on the characteristic patterns of basic psychological need satisfaction and frustration across the latent profiles and the observed characteristics of each dimension, the four profiles were named as follows: (1) Based on the maximum posterior probability assignment rule, 35 participants (8.1%) were classified into Profile 1. This group exhibited significantly low levels of satisfaction across all three basic psychological needs, coupled with moderate levels of frustration, indicating an overall state of psychological resource depletion. This profile was named the “Basic Psychological Need Deficiency type. (2) Based on the maximum posterior probability assignment rule, 199 participants (46.3%) were classified into Profile 2. This group demonstrated high levels of basic psychological need satisfaction and low levels of frustration, reflecting positive psychological adaptation. This profile was named the “Basic Psychological Need Satisfaction type. (3) Based on the maximum posterior probability assignment rule, 143 participants (33.3%) were classified into Profile 3. This group showed relatively stable levels of both need satisfaction and frustration, experiencing a certain degree of both fulfillment and frustration. This profile was named the “Basic Psychological Need Balance type. (4) Based on the maximum posterior probability assignment rule, 53 participants (12.3%) were classified into Profile 4. This group displayed the highest levels across all dimensions of both need satisfaction and frustration, representing a contradictory psychological state. This profile was named the “Basic Psychological Need Contradiction type.” (Figure 1).

### 3.3 Univariate analysis of factors associated with different latent profiles of BPNSF in community-dwelling older adults

Univariate analysis revealed statistically significant differences among the four latent profiles of community-dwelling older adults in terms of monthly income, living situation, number of chronic diseases, health status, SWLS score, DASS-21score and PSSS score (*p* < 0.05) (detailed results are presented in Table 3).

**Table 3:** Univariate analysis of factors associated with different latent profiles of BPNSF in community-dwelling older adults (*n* = 430).

Variables	Category	Class 1 (*n* = 35)	Class 2 (*n* = 199)	Class 3 (*n* = 143)	Class 4 (*n* = 53)	*x^2^/F*	*p*
Age	60–69	22 (62.86)	137 (68.84)	99 (69.23)	34 (64.15)	2.372^a^	0.882
	70–79	10 (28.57)	48 (24.12)	30 (20.98)	15 (28.30)		
	≥80	3 (8.57)	14 (7.04)	14 (9.79)	4 (7.55)		
Sex	Male	11 (31.43)	72 (36.18)	70 (48.95)	22 (41.51)	6.979^a^	0.073
	Female	24 (68.57)	127 (63.82)	73 (51.05)	31 (58.49)		
Educational level	Primary school or below	15 (42.86)	75 (37.69)	65 (45.45)	35 (66.04)	22.949^a^	0.006**
	Junior high school	9 (25.71)	47 (23.62)	43 (30.07)	11 (20.75)		
	Senior high school	6 (17.14)	37 (18.59)	21 (14.69)	5 (9.43)		
	College or above	5 (14.29)	40 (20.10)	14 (9.79)	2 (3.77)		
Monthly income	<3,000 RMB	22 (62.86)	73 (36.68)	62 (43.36)	40 (75.47)	35.965^a^	0.000**
	3,000–5,000 RMB	9 (25.71)	48 (24.12)	37 (25.87)	9 (16.98)		
	>5,000 RMB	4 (11.43)	78 (39.20)	44 (30.77)	4 (7.55)		
Marital status	Married	23 (65.71)	164 (82.41)	116 (81.12)	47 (88.68)	9.063^a^	0.17
	Unmarried	3 (8.57)	5 (2.51)	4 (2.80)	1 (1.89)		
	Divorced/widowed	9 (25.71)	30 (15.08)	23 (16.08)	5 (9.43)		
Living situation	Living with family	25 (71.43)	153 (76.88)	123 (86.01)	41 (77.36)	13.601^a^	0.034*
	Living alone	7 (20.00)	25 (12.56)	18 (12.59)	9 (16.98)		
	Elderly care institution	3 (8.57)	21 (10.55)	2 (1.40)	3 (5.66)		
Number of chronic diseases	0	13 (37.14)	80 (40.20)	71 (49.65)	37 (69.81)	26.429^a^	0.000**
	1	10 (28.57)	89 (44.72)	46 (32.17)	9 (16.98)		
	≥2	12 (34.29)	30 (15.08)	26 (18.18)	7 (13.21)		
Health status	Good	17 (48.57)	133 (66.83)	78 (54.55)	38 (71.70)	54.433^a^	0.000**
	Fair	8 (22.86)	61 (30.35)	55 (38.46)	12 (22.64)		
	Poor	10 (28.57)	5 (2.51)	10 (6.99)	3 (5.66)		
SWLS score		18.83 ± 6.65	27.65 ± 5.04	23.85 ± 4.59	33.87 ± 2.49	89.491^b^	0.000**
DASS-21score		33.94 ± 30.33	6.74 ± 14.21	50.35 ± 34.15	103.21 ± 42.41	188.767^b^	0.000**
PSSS score		44.89 ± 15.45	66.87 ± 13.14	56.34 ± 11.80	77.74 ± 12.92	64.860^b^	0.000**

Class 1: Basic Psychological Need Deficiency Type; Class 2: Basic Psychological Need Satisfaction Type; Class 3: Basic Psychological Need Balance Type; Class 4: Basic Psychological Need Contradiction Type. *p < 0.05, **p < 0.01. ^a^Chi-square test; ^b^ANOVA.

### 3.4 Multinomial logistic regression analysis of factors associated with different latent profiles of BPNSF in community-dwelling older adults

A multinomial logistic regression analysis was conducted with the four latent profiles of basic psychological need satisfaction and frustration as the dependent variable and the factors that were statistically significant in the univariate analysis as independent variables. All categorical variables were entered into the model as nominal variables, and continuous variables were entered as raw scores. The variable coding is presented in Table 4. The results indicated that monthly income, health status, living situation, SWLS score, DASS-21score and PSSS score were significant associated factors associated with the latent profiles of basic psychological need satisfaction and frustration among community-dwelling older adults (*p* < 0.05) (detailed results are presented in Table 5).

**Table 4:** Variable assignment table.

Variables	Type	Coding method
Educational level	Nominal	Reference: College or above Dummy 1: Primary school or below (1 = yes, 0 = no) Dummy 2: Junior high school (1 = yes, 0 = no) Dummy 3: Senior high school (1 = yes, 0 = no)
Monthly income	Nominal	Reference: >5,000 RMB Dummy 1: <3,000 RMB (1 = yes, 0 = no) Dummy 2: 3000–5,000 RMB (1 = yes, 0 = no)
Living situation	Nominal	Reference: Elderly care institution Dummy 1: Living with family (1 = yes, 0 = no) Dummy 2: Living alone (1 = yes, 0 = no)
Chronic diseases	Nominal	Reference: ≥2 Dummy 1: 0 (1 = yes, 0 = no) Dummy 2: 1 (1 = yes, 0 = no)
Health status	Nominal	Reference: Poor Dummy 1: Good (1 = yes, 0 = no) Dummy 2: Fair (1 = yes, 0 = no)
SWLS score	Continuous	Raw score input
DASS-21 score	Continuous	Raw score input
PSSS score	Continuous	Raw score input

**Table 5:** Multinomial logistic regression analysis of factors associated with different latent profiles of BPNSF in community-dwelling older adults (*n* = 430).

Variables	Basic psychological need deficiency type					Basic psychological need balance type					Basic psychological need contradiction type				
	*β*	Wald χ^2^	*p*	OR	95% CI	*β*	Wald χ^2^	*p*	OR	95% CI	*β*	Wald χ^2^	*p*	OR	95% CI
Constant	9.34	23.806	<0.001	-	-	2.323	2.174	0.14	-	-	−14.482	14.038	<0.001	-	-
Education level (Reference: College or above)															
Primary school or below	−0.753	0.71	0.4	0.471	0.082–2.716	0.125	0.056	0.813	1.133	0.404–3.175	0.961	0.611	0.435	2.615	0.235–29.155
Junior high school	0.135	0.024	0.878	1.144	0.204–6.41	0.491	0.906	0.341	1.635	0.594–4.498	1.911	2.371	0.124	6.763	0.594–77.04
Senior high school	0.613	0.443	0.505	1.847	0.304–11.236	0.189	0.103	0.749	1.208	0.381–3.833	0.645	0.272	0.602	1.907	0.169–21.56
Monthly income (Reference: >5,000 RMB)															
<3,000 RMB	2.008	5.598	0.018	7.45	1.411–39.325	0.028	0.005	0.944	1.029	0.469–2.257	1.304	2.079	0.149	3.684	0.626–21.68
3,000–5,000 RMB	1.42	2.762	0.097	4.136	0.775–22.065	0.171	0.159	0.69	1.187	0.511–2.755	1.247	1.47	0.225	3.481	0.463–26.152
Living situation (Reference: Elderly care institution)															
Living with family	−0.998	0.877	0.349	0.369	0.046–2.976	1.398	1.889	0.169	4.049	0.551–29.747	−3.485	8.82	0.003	0.031	0.003–0.306
Living alone	−0.884	0.565	0.452	0.413	0.041–4.142	1.05	0.947	0.331	2.858	0.345–23.686	−3.879	7.243	0.007	0.021	0.001–0.348
Number of chronic diseases (Reference: ≥2)															
0	−0.751	0.994	0.319	0.472	0.108–2.066	0.474	0.827	0.363	1.606	0.578–4.462	1.395	1.919	0.166	4.034	0.561–29.017
1	−1.311	3.193	0.074	0.269	0.064–1.135	−0.31	0.373	0.542	0.733	0.271–1.985	−0.259	0.057	0.812	0.772	0.091–6.529
Health status (Reference: Poor)															
Good	−1.371	1.878	0.171	0.254	0.036–1.804	−0.324	0.13	0.718	0.723	0.125–4.197	−1.345	0.38	0.537	0.261	0.004–18.71
Fair	−2.519	6.533	0.011	0.081	0.012–0.556	−0.3	0.116	0.734	0.741	0.131–4.178	−0.494	0.053	0.819	0.61	0.009–41.676
SWLS score	−0.232	19.873	<0.001	0.793	0.716–0.878	−0.132	13.387	<0.001	0.876	0.816–0.94	0.462	20.056	<0.001	1.588	1.297–1.944
DASS-21 score	0.04	15.955	<0.001	1.041	1.021–1.062	0.056	72.027	<0.001	1.058	1.044–1.071	0.067	54.247	<0.001	1.07	1.051–1.089
PSSS score	−0.079	16.664	<0.001	0.924	0.889–0.96	−0.033	6.499	0.011	0.968	0.944–0.992	−0.03	1.898	0.168	0.97	0.93–1.013

Referenced to the basic psychological needs satisfaction type.

## 4 Discussion

### 4.1 Heterogeneity in the characteristics of basic psychological need satisfaction and frustration in community-dwelling older adults

This study, based on latent profile analysis, identified four distinct profiles of basic psychological need satisfaction and frustration in community-dwelling older adults: the Basic Psychological Need Deficiency Type, the Basic Psychological Need Satisfaction Type, the Basic Psychological Need Balance Type, and the Basic Psychological Need Contradiction Type. This indicates significant heterogeneity in the levels of basic psychological need satisfaction and frustration within this population. Notably, this is the first study to identify the “Contradiction Type” in community-dwelling older adults, a group characterized by the simultaneous experience of high need satisfaction and high need frustration. This finding extends Basic Psychological Needs Theory by providing empirical support for the view that need satisfaction and frustration are not opposite poles of a single continuum but are relatively independent psychological constructs that can co-occur under specific circumstances.

#### 4.1.1 Basic psychological need deficiency type

Community-dwelling older adults classified as the “Basic Psychological Need Deficiency type” exhibited moderate to low levels of both basic psychological need satisfaction and frustration. This group was characterized by a high proportion of individuals with low income, low education levels, and multimorbidity, accompanied by lower satisfaction with life, lower social support, and higher emotional distress. The accumulation of multiple disadvantages is associated with a chronic unmet status of basic psychological needs. This pattern appears to coincide with higher psychological vulnerability, suggesting that this group may be at increased risk for psychological problems (Ni et al., 2024; Evans et al., 2020; Giang et al., 2024). From a practical perspective, healthcare professionals should prioritize this group for intervention by establishing individual case management files, conducting regular psychological assessments, and providing accessible psychological counseling and companionship services, gradually enhancing their level of basic psychological need satisfaction.

#### 4.1.2 Basic psychological need satisfaction type

The “Basic Psychological Need Satisfied type” constituted the largest proportion of community-dwelling older adults. This group demonstrated significant advantages, including high income, high education levels, a low burden of chronic diseases, high satisfaction with life, low emotional distress, and high social support, representing the most psychologically adaptive group within the community. Abundant material resources, favorable educational backgrounds, and good physical health enable them to continuously experience autonomy, competence, and relatedness, forming a positive psychological cycle (Chen and Zhang, 2021; Upasen et al., 2024). Intervention strategies for this population should focus on both maintenance and promotion. Communities should continuously provide rich cultural and recreational activities and platforms for autonomous participation, creating a supportive environment that sustains their positive psychological state. From a practical perspective, healthcare professionals should strengthen health management and emotional monitoring to prevent psychological fluctuations caused by sudden health events or life changes, promoting the long-term stable development of their psychological strengths.

#### 4.1.3 Basic psychological need balance type

The “Basic Psychological Need Balance type” of community-dwelling older adults exhibited intermediate levels of both basic psychological need satisfaction and frustration experiences, as well as sociodemographic characteristics, placing them between the Deprived and Satisfied types. In the multivariate analysis, this profile had few significant predictors, suggesting that its formation mechanism may be influenced by a combination of multiple factors (Brinkhof et al., 2025). These older adults may be in a dynamic stage of psychological adaptation, representing an adaptive adjustment outcome when facing physical decline and social role transitions, demonstrating considerable plasticity (Fang et al., 2025). From a practical perspective, healthcare professionals should offer mental health promotion activities such as lectures on mental health and stress management training to enhance their psychological resilience and facilitate their transition towards the “Basic Psychological Need Satisfaction type.”

#### 4.1.4 Basic psychological need contradiction type

The “Basic Psychological Need Contradiction type” represents a particularly noteworthy profile in this study. Individuals in this group simultaneously reported high levels of both need satisfaction and need frustration. It highlights the unique research value of simultaneously examining need satisfaction and frustration from a person-centered perspective. If only satisfaction or frustration were considered in isolation, this subgroup—characterized by the co-occurrence of high levels on both dimensions—might be overlooked.

This finding provides empirical support for recent theoretical developments within Basic Psychological Needs Theory, suggesting that satisfaction and frustration are not mutually exclusive but can coexist under certain conditions. Rather than interpreting this pattern as a paradox, it may reflect a complex and dynamic psychological state in which individuals experience both supportive and constraining environmental influences. Older adults may receive substantial social support that fulfills their relatedness needs, while simultaneously experiencing restrictions in autonomy or competence due to health or socioeconomic limitations. Importantly, this profile should be interpreted cautiously (Yu et al., 2022). The coexistence of high satisfaction and high frustration does not necessarily imply a stable or desirable psychological state; instead, it may indicate increased psychological tension or sensitivity to environmental conditions (Haghighat and Huo, 2022). This nuanced pattern highlights the importance of examining multiple dimensions of basic psychological needs simultaneously, rather than relying on single indicators. From a practical perspective, the findings suggest that interventions should not focus solely on increasing support, but also consider reducing sources of need frustration and addressing potential imbalances between different psychological needs.

### 4.2 Factors influencing BPNSF in community-dwelling older adults

#### 4.2.1 Monthly income

The results of this study show that community-dwelling older adults with lower monthly income were more likely to belong to the “Basic Psychological Need Deficiency type.” Economic resources serve as a crucial material foundation for meeting the basic psychological needs of older adults. Financial constraints may be associated with reduced autonomy in deciding to participate in social activities. Prolonged economic pressure is also associated with feelings of powerlessness and self-doubt, which could negatively affect perceived competence. Furthermore, a lack of economic resources can lead to the shrinking of their social networks, hindering the satisfaction of relatedness needs (Schüz et al., 2016; Zheng et al., 2024). From a practical perspective, these results suggest that interventions targeting low-income older adults may benefit from integrating psychological support with social assistance resources. Such approaches may help alleviate the negative impact of economic pressure on mental health at its source.

#### 4.2.2 Living situation

The results showed that community-dwelling older adults residing in nursing homes were more likely to belong to the “Basic Psychological Need Contradiction type.” This pattern may reflect the coexistence of supportive and restrictive aspects within institutional care settings. On the one hand, nursing homes typically provide structured care, daily assistance, and opportunities for social interaction. On the other hand, institutional management practices may limit personal choice and independence (Ferrand and Martinent, 2021). This finding may suggest that different dimensions of basic psychological needs can be differentially associated with environmental conditions. It highlights the importance of considering both supportive and constraining aspects of living environments when examining psychological experiences in older adults.

#### 4.2.3 Health status

The results of this study show that community-dwelling older adults with poorer self-rated health were more likely to belong to the “Basic Psychological Need Deficiency type.” Health status is a fundamental condition for individuals to participate in social life and exercise autonomous action. Health problems may be associated with limitations in physical function and mobility, which could make it more difficult for older adults to independently complete daily tasks they were once competent to perform. Concurrently, reduced mobility may be associated with decreased social interaction outside the home, which could contribute to gradual withdrawal from existing social networks (Tang et al., 2020; Hansson et al., 2019). From a practical perspective, healthcare professionals should regard health intervention as a foundational project for psychological promotion, paying close attention to older adults with poor self-rated health. Measures such as home modifications for aging in place can help them maintain basic self-care ability and positive health perceptions, providing essential support for the satisfaction of basic psychological needs.

#### 4.2.4 Satisfaction with life

The results of this study show that community-dwelling older adults with higher scores on satisfaction with life were more likely to belong to the “Basic Psychological Need Satisfaction type.” Satisfaction with life, as an overall cognitive evaluation of quality of life, reflects the cumulative effect of satisfying the needs for autonomy, competence, and relatedness in older adults (Shi et al., 2021). Older adults with high satisfaction with life may be better able to adapt to role transitions after retirement, may have more opportunities for autonomous decision-making in daily life, and may maintain moderate social participation, which could contribute to a positive cycle for basic psychological needs (Rouse et al., 2020). From a practical perspective, healthcare professionals should conduct mental health education promoting active aging, encourage older adults to undertake roles within their capabilities in community activities, and guide family members to foster a communicative atmosphere of respect and listening, helping older adults gain a sense of belonging and identity through emotional connections.

#### 4.2.5 Anxiety-depression-stress

The results of this study show that community-dwelling older adults with higher scores on anxiety-depression-stress were more likely to belong to the “Basic Psychological Need Contradiction type.” It is possible that older adults with higher levels of anxiety, depression, or stress are more sensitive to external stimuli. They might tend to interpret care from others as support while also perceiving everyday frustrations as threats to autonomy or competence. Such a pattern could create a subjective experience where need satisfaction and frustration co-occur, potentially maintaining a state of psychological tension (Kuang et al., 2024; Windsor et al., 2024). From a practical perspective, healthcare professionals should conduct regular mental health screenings to promptly identify community-dwelling older adults with emotional distress. Interventions such as mindfulness-based stress reduction and cognitive-behavioral therapy can be employed to alleviate their negative emotions.

#### 4.2.6 Perceived social support

The results of this study show that community-dwelling older adults with higher scores on perceived social support were more likely to belong to the “Basic Psychological Need Satisfaction type.” Higher levels of perceived social support mean that older adults perceive more abundant emotional support and practical assistance from family, friends, and significant others. The positive feedback from these external resources can effectively enhance their confidence in autonomous decision-making, alleviate the crisis of competence stemming from declining physical function, and strengthen the satisfaction of relatedness needs through interpersonal interactions (Obioha et al., 2025; Zhang and Dong, 2023). From a practical perspective, healthcare professionals can mobilize attention and support from various sectors of society for community-dwelling older adults through advocacy, guidance, and policy support, thereby providing diversified social support.

### 4.3 Limitations

This study has several limitations. First, the participants were recruited from a single city within a limited time period using the convenience sampling method, and community-level factors were not systematically investigated. Second, two latent classes had relatively small sample sizes, resulting in wide confidence intervals for some odds ratios. Third, the multinomial logistic regression was conducted using a classify-then-regress approach, which assumes that the assigned profiles are observed without error and that the covariates influence only profile membership, not within-class variation. This may lead to biased parameter estimates. Fourth, all questionnaires in this study were administered in a fixed order, which may lead to practice and fatigue effects. Future research could adopt a counterbalanced questionnaire order design, expand the sampling scope, include more diverse communities, systematically investigate community-level factors, and employ more rigorous modeling approaches to enhance the representativeness and generalizability of the findings and reduce research bias.

## 5 Conclusion

This study identified four distinct latent profiles of basic psychological need satisfaction and frustration among community-dwelling older adults through latent profile analysis. These findings indicate significant population heterogeneity in basic psychological need satisfaction and frustration within this group. The formation of these profiles is influenced by multiple factors. It is recommended that healthcare professionals implement tiered and targeted mental health services based on the distinct characteristics of basic psychological need satisfaction and frustration across different profiles, aiming to enhance their level of basic psychological need satisfaction and promote active aging.

## Data Availability

The raw data supporting the conclusions of this article will be made available by the authors, without undue reservation.
